# Liquid Biopsy and Cancer: An Ongoing Story

**DOI:** 10.3390/jcm12072690

**Published:** 2023-04-04

**Authors:** Erika Di Zazzo, Mariano Intrieri, Sergio Davinelli

**Affiliations:** 1 UOC Laboratorio Analisi, Ospedale “A. Cardarelli”, 86100 Campobasso, Italy; erika.dizazzo@unimol.it; 2 Department of Medicine and Health Sciences “V. Tiberio”, University of Molise, 86100 Campobasso, Italy; intrieri@unimol.it

The concept that body fluids may reveal the presence of disease dates back to ancient Greek history, when Hippocrates (ca. 460–370 BCE) introduced the humoral theory based on the principle that the human body consisted of four major fluids or humors (i.e., blood, phlegm, yellow bile, and black bile) that needed to be maintained in equilibrium in order to promote good health and well-being [1]. Nowadays, the most extensively studied body fluids are blood and urine, but many other biological liquids, such as cerebrospinal liquid, seminal fluid, and saliva, may offer great diagnostic and prognostic potential. Despite advances in therapeutics and molecular profiling technologies, cancer continues to be one of the major causes of mortality worldwide. The molecular profile of cancer is currently assessed by resected or biopsied tumors; however, tissue biopsy has inherent limitations. Indeed, this sampling approach is now regarded as inadequate and unreliable to detect spatial and temporal heterogeneity of the tumor [2,3]. Practical and scientific issues of tissue sampling have led to increased interest in the development of minimally invasive liquid biopsies to capture the dynamic nature of cancer through its evolution [4]. In cancer, the term liquid biopsy refers to the analysis of tumor-specific markers from various biological fluids, usually blood. Several techniques used to detect circulating tumor DNA (ctDNA) can be applied to many body fluids, including blood, to identify novel oncologic biomarkers for diagnosis, prognosis, and monitoring of therapeutic response [5]. Historically, studies of disseminated tumor cells in bone marrow and circulating tumor cells (CTCs) in peripheral blood have provided crucial insights into cancer biology and the metastatic process. Consistent with this trend, the new field of precision oncology is mainly focused on cancer biomarkers derived from primary tumor or metastatic site components that circulate in the bloodstream. Blood of cancer patients may contain many types of biological materials released by tumor cells, including CTCs, cell-free DNA (cfDNA), RNA (mRNA and miRNA) extracellular vesicles (EVs), and exosomes [6]. For example, the most recent literature on gastric cancer supports a prognostic role of CTCs in all the disease stages. Moreover, the cfDNA and ctDNA detection may inform us regarding the risk of gastric cancer recurrence and metastatic dissemination. There is also evidence that the presence of specific gastric-cancer-derived exosomal miRNA may contribute resistance to chemotherapy [7]. Currently, liquid biopsy also represents a powerful tool to design tailored interventions in lung cancer. ctDNA assays can be used to genotype different lung tumor types and monitor the impact of genotype-directed therapies. Indeed, liquid biopsy kits for oncogenic driver mutations, such as those of *EGFR* and *ALK*, have already been approved for clinical use [8]. ctDNA/cfDNA analysis is applied for the screening of therapeutic targets, to unveil drug resistance mechanisms, and the monitoring of minimal residual disease (MRD) in non-small-cell lung cancer (NSCLC) with the *EGFR* mutation [9]. After lung cancer, prostate cancer is the second-most-frequent malignancy in men worldwide. Prostate cancer pathogenesis relies on the androgen/androgen receptor (AR) circuit, and progression is frequently depicted by a deregulated AR-mediated signaling activation or AR variants. Recently, Young et al. developed two multiplexed droplet digital PCR (ddPCR) assays to detect AR copy number and the key point mutation T877A. These assays cross validate each other to produce reliable AR amplification and mutation data from plasma cfDNA of advanced prostate cancer patients [10]. Facilitated by the rapid development of next-generation sequencing (NGS) technologies, cfDNA has shown utility for the management of several cancer types, including colorectal cancer. Rodríguez-Casanova et al. used an NGS panel (TruSight Tumor 170) to identify gene variants in cfDNA from a cohort of 20 metastatic patients affected by colorectal cancer. They detected variants with clinical significance in *KRAS* and *PIK3CA* genes, providing an assay that could be easily implemented for detecting somatic alterations [11]. Moreover, an NGS liquid biopsy analysis of cfDNA variants may also allow for novel classifications with relevant diagnostic and therapeutic implications [12]. The assessment of genes involved in the angiogenic process may help to find new diagnostic biomarkers and improve the response to antiangiogenic therapy. A prospective analysis of peripheral blood samples from 59 metastatic colorectal cancer patients showed that *PTGS2*, *GUCY2C,* and *JAG1* upregulation correlated with high discrimination ability between metastatic colorectal cancer patients and healthy donors. Although these biomarkers were not correlated with overall survival, *GUCY2C* and *GUCY2C/PTGS2* expression correlated significantly with the response to antiangiogenic agents [13]. It is well known that miRNAs play a central role in various cellular processes implicated in the development and progression of NSCLC. A recent study confirmed that miR-335 and miR let-7a expression levels in the serum of patients with NSCLC correlated with lymph node metastasis. Therefore, these miRNAs may potentially serve as non-invasive molecular biomarkers to predict metastasis in liquid biopsy [14]. In recent years, PIWI-interacting RNAs (piRNAs) are emerging as crucial players in cancer genomics. An increasing number of studies have shown that aberrant piRNA expression is a signature feature across multiple tumor types. A novel piRNA, known as *piR-162725*, was recently identified in pancreatic cancer patients by liquid biopsy. Interestingly, the potential of the serum carbohydrate antigen 19-9 (CA19-9) biomarker to identify pancreatic cancer patients was greatly enhanced when combined with *piR-162725* detection [15]. Given that alterations in DNA or in different RNA species could not reveal what happened in proteins, proteome profiling in liquid biopsy samples may provide clinically relevant information throughout all stages of cancer progression. Using a one-dimensional liquid chromatography–mass spectrometry (1D LC-MS/MS) approach, a pilot study assessed the feasibility of nipple aspirate fluid (NAF) as a liquid biopsy sample for the investigation of breast health. This proof-of-principle study revealed that a rapid LC-MS method can quantify changes reflected in the NAF proteome associated with breast cancer development [16]. Collectively, these data continue to suggest that liquid biopsy is more feasible, less invasive, and more comprehensive than tissue biopsy to evaluate tumor heterogeneity. It is, therefore, reasonable to assume that, once methodological procedures will be harmonized across laboratories, liquid biopsy will lead to a profound change in the oncology practice.
